# Supplementary material for: Genetic factors associated with suicidal behaviors and alcohol use disorders in an American Indian population
Source: Mol Psychiatry. 2024 Jan 4;29(4):902–13. doi: 10.1038/s41380-023-02379-3 (PMC11176067; doi:10.1038/s41380-023-02379-3)
Supplement: Supplementary file 1 — Supplementary Information [file 41380_2023_2379_MOESM1_ESM.docx]

Table of Content

[Supplementary Figures 3](#_Toc149958063)

[Figure S1. AUD severity score is highly aligned and correlated with DSM5 AUD diagnosis in AI. 3](#_Toc149958064)

[Figure S2. AUD severity score is positively correlated with suicidal behaviors in AI. 4](#_Toc149958065)

[Figure S3: Statistical powers 5](#_Toc149958066)

[Supplementary Tables 6](#_Toc149958067)

[Table S1. Demographics of the American Indian cohort. 6](#_Toc149958068)

[Table S2. Alcohol-related life events in the clinical course of AUD. 7](#_Toc149958069)

[Table S3. Determine covariates to be included for AUD severity and suicidal behaviors genetic association analysis in a main model and an extended model incorporating socioeconomic factors. 8](#_Toc149958070)

[Table S4. Brain eQTLs for rs184204326 on gene *FBXO11* that is significantly associated with SB-AUD in AI. 10](#_Toc149958071)

[Table S5.Top variants from bivariate GWAS between SB and AUD severity or DSM5 AUD diagnosis. 11](#_Toc149958072)

[Table S6. Gene sets enriched in the top genes associated with SB-AUD. 13](#_Toc149958073)

[Table S7. Top genes with rare and low-frequency variants associated with SB in AI using extended model. 15](#_Toc149958074)

[Table S8. Top pathways and gene sets with rare and low-frequency nonsynonymous variants associated with SB in AI using extended model. 15](#_Toc149958075)

[Table S9. Functional networks associated with genes whose rare variants were significantly associated with suicidal behaviors (FDR<0.1). 16](#_Toc149958076)

[Table S10a. Top genes with rare and low-frequency variants associated with SB-AUD in AI using the main model. 17](#_Toc149958077)

[Table S10b. Top pathways with rare and low-frequency variants associated with SB-AUD in AI using the main model. 17](#_Toc149958078)

[Supplementary Material and Method 18](#_Toc149958079)

[S1. Participants 18](#_Toc149958080)

[S2. AUD severity phenotype 18](#_Toc149958081)

[S3. Genotyping of American Indian cohort 18](#_Toc149958082)

[S4. Rare and low frequency variant analysis 19](#_Toc149958083)

[S5. Pathway database used in rare-and-low-frequency variant test 19](#_Toc149958084)

[S6. Meta-analysis of SB and AUD rare-and-low-frequency variant tests 19](#_Toc149958085)

[S7. Functional analysis with FUMA 20](#_Toc149958086)

[Supplementary Reference 21](#_Toc149958087)

# **Supplementary Figures**

## Figure S1. AUD severity score is highly aligned and correlated with DSM5 AUD diagnosis in AI.

Spearman’s rank correlation = 0.91

Pearson’s correlation = 0.87


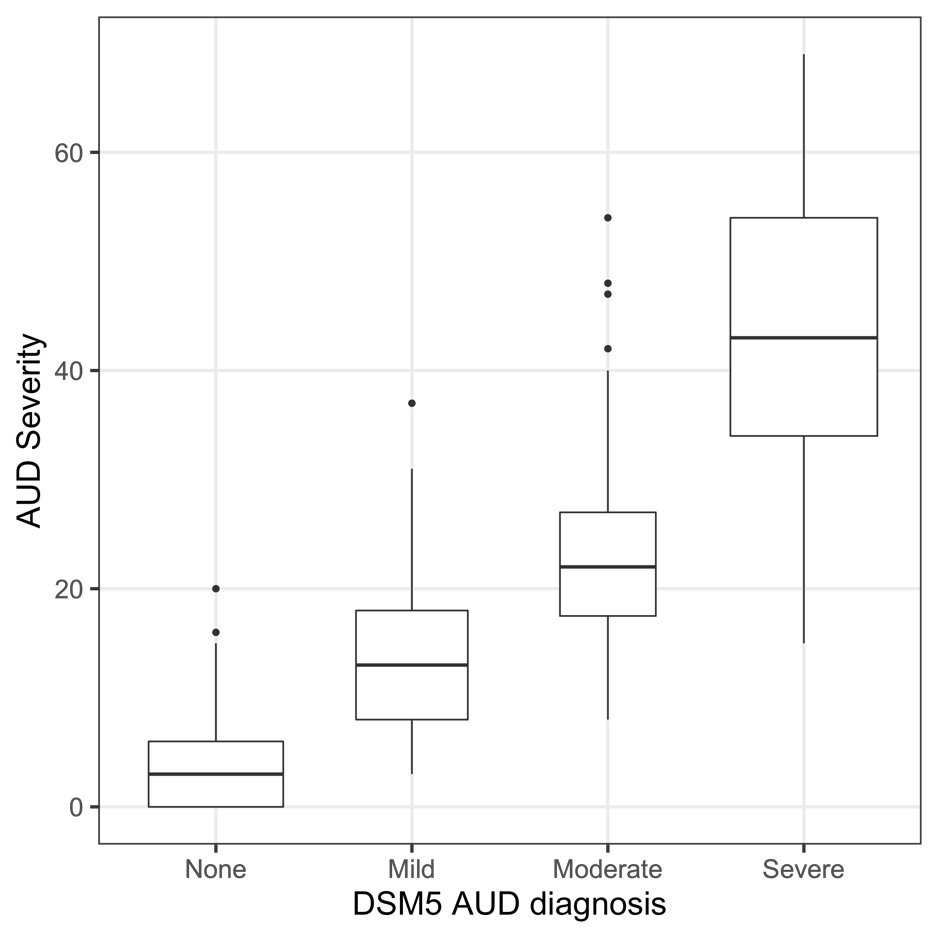


## Figure S2. AUD severity score is positively correlated with suicidal behaviors in AI.

Spearman’s rank correlation = 0.26

Pearson’s correlation = 0.29


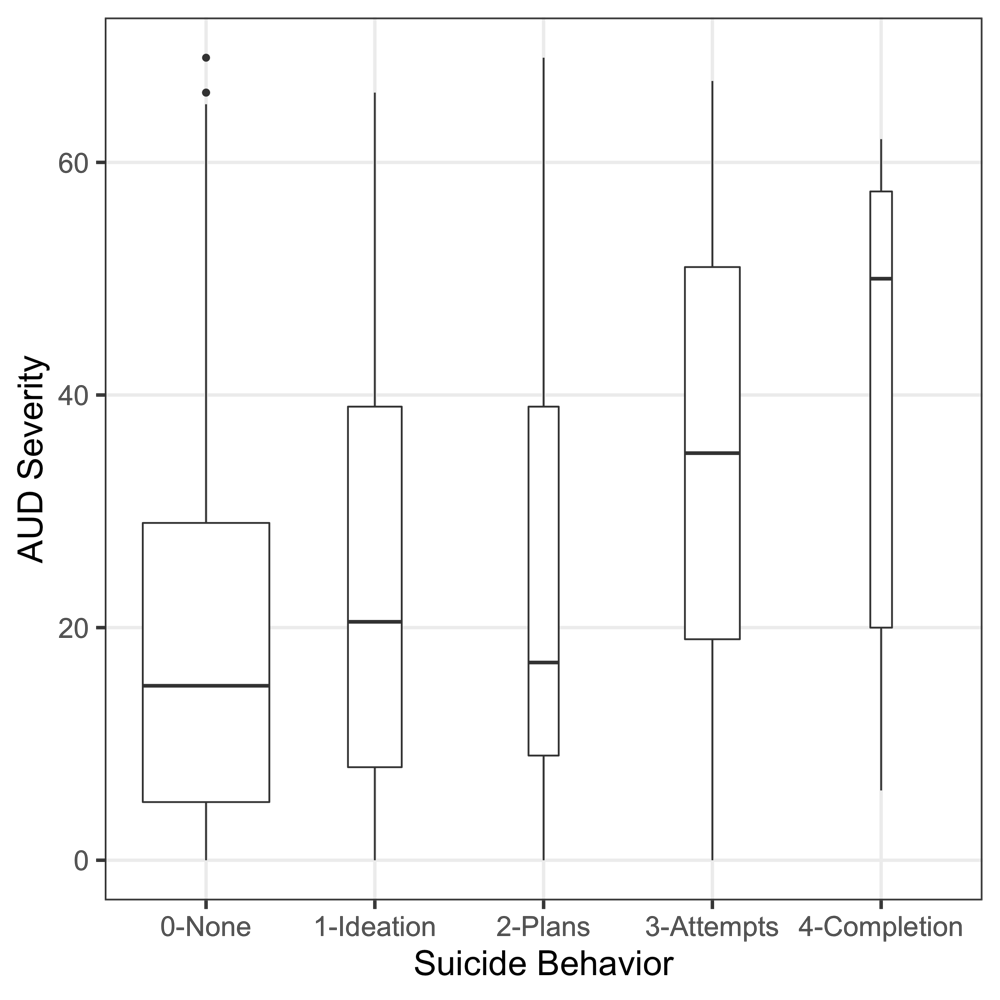


## Figure S3: Statistical powers

A: Statistical power as a function of variance explained by the marker (effect size) to detect genomic association with sample size *n* = 742. B: Desired sample size to detect association for given effect sizes in order to achieve 80% statistical power. The significant level or type I error rate is set at 5×10^−8^ (typical GWAS significant threshold) and 5×10^−7^ (suggestive significance). The power calculation was done using the Genetic Power Calculator [Purcell et al., 2003]. Only additive effects were considered. The samples were assumed unrelated. The relatedness in the samples generally yield slightly increased powers [Sham and Purcell, 2014].

A.


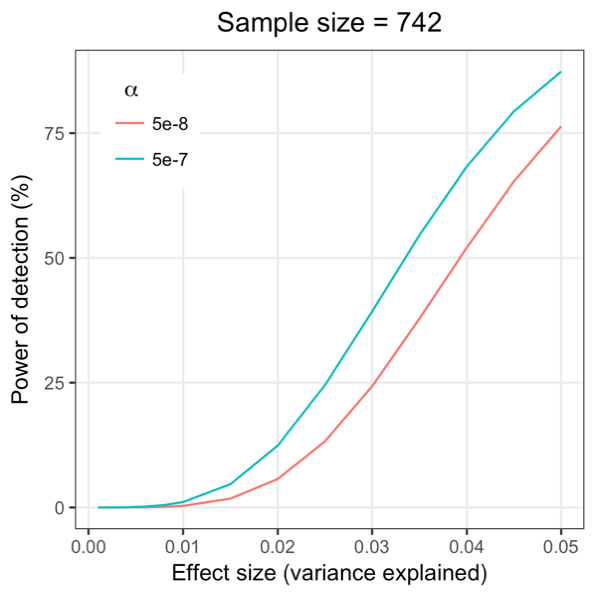


B.


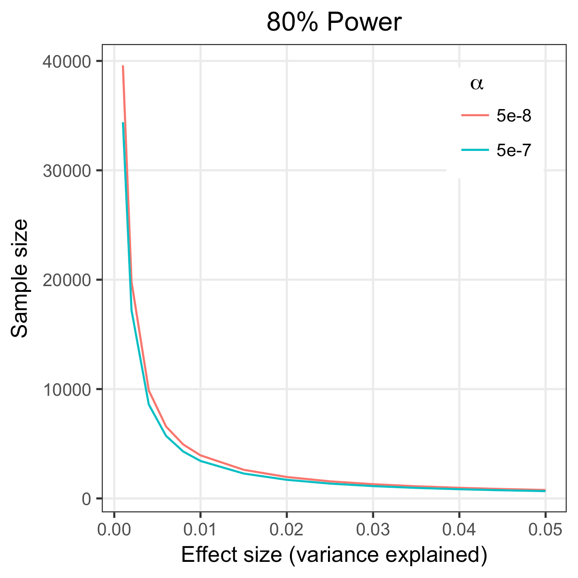


# **Supplementary Tables**

##

## Table S1. Demographics of the American Indian cohort.

| Cohort | American Indian (AI) |
| --- | --- |
| Individuals (*n*) | 743 |
| Family | 170 |
| Gender | M: 317, F: 426 |
| Age^1^ (yrs) | 31.2±13.2 [18, 82] |
| Education^1^ (yrs) | 11.6±1.6 [3, 17] |
| Employed(No\|Yes) | 458\|266 |
| Religion (N\|A\|C1\|C2)^2^ | 149\|53\|209\|328 |
| Ethnicity | Native American |

^1^ In the format of mean ± standard deviation [min, max].

^2^ Religion: N=None, A=American Indian religions, C1=Christianity (non-Catholic), C2=Catholicism.

## Table S2. Alcohol-related life events in the clinical course of AUD.

The order of the events was based on the mean age of the occurrence with the first event happening earliest and the last (36^th^) event occurring latest in a lifetime [Ehlers et al., 2004]. Severity weights that were assigned to events were used to compute AUD severity level [Peng et al., 2019].

| Severity weight 1 | Severity weight 2 | Severity weight 3 |
| --- | --- | --- |
| 1. Arguments | 13. Binges | 25. Arrested for alcohol related behavior |
| 2. Physical fights | 14. Tolerance | 26. Problems in love relationship |
| 3. Problems at work/school | 15. Interfered with work | 27. Considered excessive drinker |
| 4. Problems with family, friends | 16. Self injury while drunk | 28. Guilt |
| 5. Hitting others without fighting | 17. Decreased important activities | 29. Wanted to quit 3+ times |
| 6. Objections from family, friends | 18. Inability to change drinking behavior | 30. Withdrawal |
| 7. Drank while in hazardous situations | 19. Morning drinking | 31. Unable to quit/cut down |
| 8. Drank when not intended | 20. Drank more than intended | 32. Arrested for DUI |
| 9. Lost friends | 21. Used rules for drinking | 33. Shakes |
| 10. Blackouts | 22. Little time for non-drinking activities | 34. Continues despite health problems |
| 11. Hit/threw things | 23. Strong desire for alcohol | 35. Health problems occurred |
| 12. Hit family member | 24. Psychological impairment | 36. Sought professional help |

## Table S3. Determine covariates to be included for AUD severity and suicidal behaviors genetic association analysis in a main model and an extended model incorporating socioeconomic factors.

As shown in Table S3A for AUD severity and Table S3B for SB, the main model included sex, age, and age squared as covariates for all analyses, even though SB was not significantly associated with these three variables in the AI. As shown in column “Extended Model 1”, the extended model initially included sex, age, age squared, gross income, years of education, employment status, marriage status, religion (none, American Indian, Christianity (non-Catholic), Catholicism), and religious service attended. Since only American Indian religion was significantly associated with SB, we collapsed the religion variable into a binary trait: American Indian religion vs all others, resulting in the “Extended Model 2”. We then dropped the factors that were negligible for AUD and SB with *p*>0.1 from the extended model 2 to obtain the final extended model for subsequent analyses. As a result, in addition to the covariates in the main model, the extended model included years of education, currently employed, and religion as covariates for the SB-AUD bivariate analysis, and years of education and religion for the SB rare and low-frequency variant analyses. Due to missing data, with all socioeconomic factors listed in the tables, the sample size dropped from 742 to 686 for AUD, and from 743 to 687 for SB. Once the factors with negligible effects were dropped, the sample size in the final extended model was 720 for the SB-AUD bivariate analysis, and 740 for the SB rare and low-frequency variant analysis.

| (A) AUD severity | Main Model (*N*=742) | | | | Extended Model 1 (*N*=686) | | | | Extended Model 2 (*N*=686) | | | |
| --- | --- | --- | --- | --- | --- | --- | --- | --- | --- | --- | --- | --- |
|  | β | SE | χ^2^ | *p*-value | β | SE | χ^2^ | *p*-value | β | SE | χ^2^ | *p*-value |
| (Intercept) | 8.37 | 4.19 | 2.00 | 0.05 | 11.82 | 6.99 | 1.69 | 0.09 | 11.47 | 6.86 | 1.67 | 0.09 |
| Sex | 6.09 | 1.33 | 4.59 | 5.14E-06 | 6.33 | 1.39 | 4.55 | 6.36E-06 | 6.26 | 1.38 | 4.52 | 7.21E-06 |
| Age | 2.00 | 0.24 | 8.29 | 5.45E-16 | 2.10 | 0.27 | 7.85 | 1.65E-14 | 2.09 | 0.27 | 7.84 | 1.79E-14 |
| Age-squared | 0.02 | 0.00 | 7.83 | 1.69E-14 | 0.03 | 0.00 | 7.46 | 2.64E-13 | 0.03 | 0.00 | 7.43 | 3.20E-13 |
| Gross income |  |  |  |  | 0.18 | 0.31 | 0.58 | 0.56 | 0.17 | 0.31 | 0.53 | 0.59 |
| Years of education |  |  |  |  | 1.78 | 0.46 | 3.88 | 1.14E-04 | 1.72 | 0.45 | 3.80 | 1.57E-04 |
| Currently employed |  |  |  |  | 2.71 | 1.46 | 1.85 | 0.06 | 2.72 | 1.46 | 1.87 | 0.06 |
| Married |  |  |  |  | 3.00 | 1.97 | 1.52 | 0.13 | 3.10 | 1.97 | 1.58 | 0.12 |
| Religion (American Indian) |  |  |  |  | 5.51 | 3.04 | 1.81 | 0.07 | 5.32 | 2.63 | 2.02 | 0.04 |
| Religion (Christianity) |  |  |  |  | 0.65 | 2.14 | 0.31 | 0.76 |  |  |  |  |
| Religion (Catholicism) |  |  |  |  | 0.81 | 1.92 | 0.42 | 0.67 |  |  |  |  |
| Religious services attended |  |  |  |  | 0.03 | 0.02 | 1.56 | 0.12 | 0.03 | 0.02 | 1.45 | 0.15 |

| (B) Suicidal Behaviors (SB) | Main Model (*N*=743) | | | | Extended Model 1 (*N*=687) | | | | Extended Model 2 (*N*=687) | | | |
| --- | --- | --- | --- | --- | --- | --- | --- | --- | --- | --- | --- | --- |
|  | β | SE | χ^2^ | *p*-value | β | SE | χ^2^ | *p*-value | β | SE | χ^2^ | *p*-value |
| (Intercept) | 0.30 | 0.27 | 1.13 | 0.26 | 1.39 | 0.45 | 3.10 | 2.03E-03 | 1.40 | 0.44 | 3.17 | 1.60E-03 |
| Sex | 0.11 | 0.09 | 1.30 | 0.19 | 0.13 | 0.09 | 1.47 | 0.14 | 0.13 | 0.09 | 1.50 | 0.14 |
| Age | 0.02 | 0.02 | 1.17 | 0.24 | 0.02 | 0.02 | 1.03 | 0.30 | 0.02 | 0.02 | 1.01 | 0.31 |
| Age-squared | 0.00 | 0.00 | 1.15 | 0.25 | 0.00 | 0.00 | -1.06 | 0.29 | 0.00 | 0.00 | 1.03 | 0.30 |
| Gross income |  |  |  |  | 0.01 | 0.02 | 0.30 | 0.77 | 0.01 | 0.02 | 0.33 | 0.74 |
| Years of education |  |  |  |  | -0.10 | 0.03 | -3.39 | 7.49E-04 | 0.10 | 0.03 | 3.36 | 8.33E-04 |
| Currently employed |  |  |  |  | -0.06 | 0.09 | -0.61 | 0.54 | 0.06 | 0.09 | 0.61 | 0.54 |
| Married |  |  |  |  | -0.02 | 0.13 | -0.16 | 0.87 | 0.02 | 0.13 | 0.19 | 0.85 |
| Religion (American Indian) |  |  |  |  | 0.58 | 0.20 | 2.92 | 3.68E-03 | 0.55 | 0.17 | 3.22 | 1.35E-03 |
| Religion (Christianity) |  |  |  |  | 0.00 | 0.14 | 0.00 | 1.00 |  |  |  |  |
| Religion (Catholicism) |  |  |  |  | 0.05 | 0.13 | 0.43 | 0.66 |  |  |  |  |
| Religious services attended |  |  |  |  | 0.00 | 0.00 | 1.55 | 0.12 | 0.00 | 0.00 | 1.55 | 0.12 |

## Table S4. Brain eQTLs for rs184204326 on gene *FBXO11* that is significantly associated with SB-AUD in AI.

*P*-values are listed for each brain tissue available in the Braineac database. CRBL-Cerebellar cortex, FCTX-Frontal cortex, HIPP-Hippocampus, MEDU-Medulla (inf. olivary nucleus), OCTX-Occipital cortex, PUTM-Putamen, SNIG-Substantia nigra, TCTX: Temporal cortex, THAL-Thalamus, WHMT-Intralobular white matter, ALL averaged-Averaged over all brain tissues.

Bond font: significant eQTL (*p*-value < 0.05).

| **GENE** | **CRBL** | **FCTX** | **HIPP** | **MEDU** | **OCTX** | **PUTM** | **SNIG** | **TCTX** | **THAL** | **WHMT** |
| --- | --- | --- | --- | --- | --- | --- | --- | --- | --- | --- |
| *EPCAM* | 1.90E-01 | 4.90E-01 | 9.70E-01 | 7.80E-01 | 5.00E-01 | **1.90E-03** | 2.50E-01 | 9.00E-01 | 4.40E-01 | 9.50E-02 |
| *MCFD2* | 6.40E-02 | 9.80E-01 | 6.90E-01 | 2.80E-01 | **3.00E-02** | **9.20E-03** | **1.60E-02** | 6.00E-01 | **1.40E-02** | 8.10E-01 |
| *FBXO11* | 3.70E-01 | 4.90E-01 | 6.70E-01 | **3.20E-02** | 1.10E-01 | **1.20E-02** | 1.10E-01 | 2.80E-01 | **1.20E-02** | 7.60E-01 |
| *LOC644093* | 2.40E-01 | 8.70E-01 | 7.10E-01 | 7.50E-02 | 3.60E-01 | **1.70E-02** | 4.00E-01 | 8.70E-01 | 3.70E-01 | 8.30E-01 |
| *MSH2* | 4.20E-01 | 4.20E-01 | 5.10E-01 | 6.90E-02 | 1.50E-01 | **1.90E-02** | 1.20E-01 | 1.70E-01 | **1.10E-02** | 5.20E-01 |
| *MSH6,LOC440864* | 4.40E-01 | 6.60E-01 | 5.50E-01 | 1.50E-01 | 9.30E-02 | **3.40E-02** | 1.00E+00 | 9.20E-01 | 1.50E-01 | 3.70E-01 |
| *CALM2,C2orf61* | 1.10E-01 | 2.50E-01 | 7.50E-01 | 2.40E-01 | 3.30E-01 | **4.30E-02** | 6.20E-02 | 9.40E-01 | 7.00E-02 | 5.90E-01 |
| *KCNK12* | 1.40E-01 | 1.10E-01 | 9.10E-01 | 1.40E-01 | 8.60E-01 | **4.90E-02** | **1.70E-02** | 1.10E-01 | **4.50E-02** | 7.90E-01 |
| *C2orf61* | 4.90E-01 | 3.40E-01 | 5.90E-01 | 4.40E-01 | 2.40E-01 | 7.80E-02 | 9.20E-01 | 1.60E-01 | 2.80E-01 | 5.80E-01 |
| *LHCGR* | 9.90E-01 | 5.50E-01 | 8.40E-02 | 8.00E-01 | 5.10E-01 | 8.30E-02 | 1.50E-02 | 1.60E-02 | 2.60E-01 | 7.80E-01 |
| *KLRAQ1* | 4.50E-01 | 2.00E-01 | 1.70E-01 | 3.80E-01 | 5.10E-01 | 9.10E-02 | 3.10E-01 | 3.10E-01 | 2.50E-01 | 1.80E-01 |
| *LOC440863* | 6.80E-01 | 4.20E-01 | 1.20E-01 | 5.60E-01 | **2.40E-02** | 1.90E-01 | 1.80E-02 | 2.50E-01 | 6.90E-02 | 8.00E-01 |
| *LOC644093* | 1.90E-01 | 6.10E-01 | 4.50E-01 | 7.30E-01 | **3.30E-02** | 2.80E-01 | 5.80E-01 | 4.60E-02 | 1.20E-01 | 4.80E-01 |
| *STON1,GTF2A1L,*  *FLJ46838,STON1-GTF2A1L* | 8.80E-02 | 5.10E-01 | 3.40E-01 | 5.40E-02 | 5.80E-02 | 3.20E-01 | 3.50E-01 | 8.20E-01 | 8.60E-01 | 1.90E-01 |
| *FOXN2* | 8.10E-01 | 2.10E-01 | 7.80E-01 | 2.80E-01 | 9.70E-01 | 3.30E-01 | 4.40E-01 | 5.60E-01 | 7.20E-01 | 4.10E-01 |
| *TTC7A* | 2.20E-01 | 4.60E-01 | 9.00E-01 | 2.30E-01 | 1.20E-01 | 3.30E-01 | 8.60E-01 | 4.80E-01 | 7.00E-02 | 5.80E-01 |
| *LOC644093* | 8.50E-01 | 4.90E-01 | 7.10E-01 | 4.10E-01 | 4.40E-01 | 5.80E-01 | 5.70E-01 | 8.40E-01 | 9.60E-01 | 9.80E-01 |
| *C2orf61* | 9.20E-02 | 4.70E-01 | 7.60E-01 | 2.00E-01 | 3.00E-01 | 6.20E-01 | 6.70E-01 | 5.60E-01 | 2.60E-01 | 9.60E-01 |

## Table S5.Top variants from bivariate GWAS between SB and AUD severity or DSM5 AUD diagnosis.

We conducted bivariate GWAS analysis for SB and AUD severity phenotype, first using the default main model and then using the extended model for covariates. As a comparison, we also conducted bivariate analysis using SB and DSM5 AUD diagnosis phenotype (none, mild, moderate, severe AUD), using both covariate models. The AUD severity phenotype derived from AUD clinical course offered increased power for detection. The table lists the top 10 variants from each analysis and covariate model, sorted by the *p*-value from SB+AUD severity main model analysis.

| Chr | Position | Ref | Alt | dbSNP | Nearest Gene | Location | Distance to gene | MAF  AI | MAF-1000 Genomes | CADD  C-score | SB + AUD severity  *p-*value | | SB + DSM5 AUD  *p*-value | |
| --- | --- | --- | --- | --- | --- | --- | --- | --- | --- | --- | --- | --- | --- | --- |
|  |  |  |  |  |  |  |  |  |  |  | Main | Extended | Main | Extended |
| 13 | 100709891 | C | T | rs1168770030 | *ZIC2-PCCA* | Intergenic | 31446 | 0.024 | N/A | 1.19 | **2.87E-09** | 3.57E-07 | 8.22E-08 | 3.69E-06 |
| 4 | 163743605 | G | T | rs200577368 | *NAF1-FSTL5* | Intergenic | 304255 | 0.026 | <0.01 | 0.02 | **3.17E-09** | 6.00E-08 | 1.11E-06 | 8.21E-06 |
| 5 | 178238561 | G | A | rs76300969 | *AACSL* | Intron | 0 | 0.01 | 0.01 | 10.03 | **4.16E-09** | NA | **8.48E-09** | NA |
| 8 | 41731811 | A | C | rs530542541 | *ANK1* | Intron | 0 | 0.044 | 0.05 | 0.76 | **2.22E-08** | 7.78E-07 | 3.25E-07 | 9.20E-06 |
| 2 | 48105656 | G | C | rs184204326 | *FBXO11* | Intron | 0 | 0.066 | 0.08 | 0.28 | **3.63E-08** | 3.02E-07 | 1.49E-07 | 1.16E-06 |
| 9 | 22528934 | C | T | rs79833306 | *DMRTA1* | Downstream | 76462 | 0.031 | <0.01 | 13.32 | 6.75E-08 | 1.65E-07 | 1.53E-04 | 2.99E-04 |
| 1 | 169344460 | C | T | rs552582317 | *BLZF1* | Intron | 0 | 0.035 | 0.065 | 5.71 | 1.06E-07 | 2.77E-06 | 1.28E-05 | 1.15E-04 |
| 17 | 1674434 | C | G | rs1804145 | *SERPINF1* | Exon | 0 | 0.011 | 0.01 | 22.80 | 1.12E-07 | 3.50E-07 | 7.47E-07 | 2.71E-06 |
| 17 | 1683043 | C | T | rs12951668 | *SMYD4* | 3UTR | 0 | 0.011 | 0.01 | 2.18 | 1.12E-07 | 3.50E-07 | 7.47E-07 | 2.71E-06 |
| 14 | 84263845 | C | T | rs142827459 | *BX248253* | Downstream | 1727632 | 0.028 | 0.02 | 0.44 | 1.58E-07 | 5.69E-06 | 2.26E-07 | 7.37E-06 |
| 14 | 84265714 | A | G | rs147592518 | *BX248253* | Downstream | 1725763 | 0.028 | 0.02 | 13.94 | 1.58E-07 | 5.69E-06 | 2.26E-07 | 7.37E-06 |
| 11 | 40279173 | T | C | rs1478967112 | *LRRC4C* | Intron | 0 | 0.01 | N/A | 3.45 | 2.34E-07 | **2.17E-08** | 1.82E-04 | 4.04E-05 |
| 16 | 51029186 | C | T | rs78299226 | *SALL1* | Downstream | 140700 | 0.04 | 0.03 | 2.71 | 2.66E-07 | 6.43E-08 | 2.80E-07 | 9.10E-08 |
| 9 | 22528925 | A | T | rs1587692615 | *DMRTA1* | Downstream | 76453 | 0.011 | N/A | 12.93 | 2.96E-07 | 1.06E-07 | 2.26E-05 | 1.36E-05 |
| 1 | 215639418 | A | T | rs145751732 | *KCTD3* | Upstream | 101317 | 0.014 | 0.01 | 1.06 | 3.42E-07 | 3.17E-07 | 1.19E-05 | 6.43E-05 |
| 1 | 215684926 | G | A | rs72739250 | *KCTD3* | Upstream | 55809 | 0.014 | 0.01 | 0.19 | 3.42E-07 | 3.17E-07 | 1.19E-05 | 6.43E-05 |
| 19 | 42145509 | T | C | rs147081270 | *CEACAM4* | Upstream | 12067 | 0.012 | <0.01 | 1.45 | 3.60E-07 | 8.64E-07 | 1.30E-07 | 2.95E-07 |
| 5 | 171526748 | G | A | rs77743512 | *STK10* | Intron | 0 | 0.019 | 0.02 | 1.24 | 4.26E-07 | 8.53E-08 | 3.47E-07 | 1.07E-07 |
| 3 | 135454986 | T | C | rs114857455 | *PPP2R3A* | Upstream | 229581 | 0.017 | 0.04 | 5.15 | 4.35E-07 | 2.05E-06 | 1.22E-07 | 7.08E-07 |
| 11 | 12078926 | T | C | rs12804449 | *MICAL2* | Upstream | 36617 | 0.106 | 0.17 | 0.79 | 4.63E-07 | 3.34E-07 | 1.59E-07 | 1.46E-07 |
| 16 | 51004663 | T | A | rs76606215 | *SALL1* | Downstream | 165223 | 0.037 | 0.01 | 1.28 | 5.31E-07 | 2.05E-07 | 5.82E-07 | 2.28E-07 |
| 3 | 135453274 | G | T | rs114548525 | *PPP2R3A* | Upstream | 231293 | 0.018 | 0.04 | 3.49 | 7.21E-07 | 3.38E-06 | 2.36E-07 | 1.32E-06 |
| 3 | 135450924 | T | C | rs6772393 | *PPP2R3A* | Upstream | 233643 | 0.018 | 0.04 | 1.55 | 7.21E-07 | 3.38E-06 | 2.36E-07 | 1.32E-06 |
| 3 | 135451341 | T | C | rs114508619 | *PPP2R3A* | Upstream | 233226 | 0.018 | 0.04 | 1.87 | 7.21E-07 | 3.38E-06 | 2.36E-07 | 1.32E-06 |
| 20 | 12900769 | G | T | rs75148426 | *SPTLC3* | Upstream | 88858 | 0.015 | 0.01 | 2.01 | 8.70E-07 | 2.29E-07 | 1.23E-06 | 4.04E-07 |
| 21 | 37866324 | A | G | rs2776290 | *CLDN14* | Intron | 0 | 0.056 | 0.15 | 0.73 | 3.35E-06 | 3.67E-07 | 1.41E-06 | 1.09E-07 |
| 21 | 37866210 | C | T | rs2850115 | *CLDN14* | Intron | 0 | 0.056 | 0.17 | 1.33 | 4.82E-06 | 5.35E-07 | 1.89E-06 | 1.49E-07 |
| 2 | 226812506 | A | T | rs13403085 | *BC017935* | Upstream | 195004 | 0.078 | 0.17 | 10.97 | 8.04E-06 | 6.81E-07 | 6.92E-06 | 3.57E-07 |
| 1 | 229190243 | T | C | rs587950 | *AX748369* | Upstream | 137289 | 0.29 | 0.37 | 1.76 | 1.51E-04 | 8.40E-05 | 6.35E-07 | 4.27E-07 |

## Table S6. Gene sets enriched in the top genes associated with SB-AUD.

| **Gene Set** | ***N***  **genes** | ***N***  **overlap** | ***P*-value** | **Adjusted**  ***P*-value** | **Genes** |
| --- | --- | --- | --- | --- | --- |
| **microRNA targets (3)** | | | | | |
| TGCAAAC MIR452 | 109 | 3 | 8.04E-05 | 1.78E-02 | *SALL1:KCNMB2:ANK1* |
| CTACTGT MIR199A | 181 | 3 | 3.59E-04 | 3.49E-02 | *LRRC4C:KCNMB2:RREB1* |
| TGTGTGA MIR377 | 199 | 3 | 4.73E-04 | 3.49E-02 | *NEGR1:HDAC9:ATXN7L1* |
| **Transcription Factor (TF) targets (27)** | | | | | |
| FOX Q2 | 213 | 4 | 2.06E-05 | 9.61E-03 | *HMCN1:FBXO11:RREB1:ATXN7L1* |
| TAATTA CHX10 01 | 823 | 6 | 3.15E-05 | 9.61E-03 | *SALL1:NPAS2:CLSTN2:RREB1:ATXN7L1:DMRTA1* |
| WGTTNNNNNAAA UNKNOWN | 555 | 5 | 5.84E-05 | 1.19E-02 | *HMCN1:NPAS2:PPP2R3A:RREB1:HDAC9* |
| CTTTAAR UNKNOWN | 990 | 6 | 8.78E-05 | 1.34E-02 | *HMCN1:SALL1:SERPINF1:FBXO11:SPTLC3:PPP2R3A* |
| YATGNWAAT OCT C | 365 | 4 | 1.66E-04 | 2.03E-02 | *SALL1:PPP2R3A:HDAC9:ATXN7L1* |
| TAAWWATAG RSRFC4 Q2 | 172 | 3 | 3.09E-04 | 2.57E-02 | *SALL1:FBXO11:PPP2R3A* |
| FOXJ2 01 | 187 | 3 | 3.95E-04 | 2.57E-02 | *HMCN1:FBXO11:ATXN7L1* |
| HNF3 Q6 | 192 | 3 | 4.26E-04 | 2.57E-02 | *FBXO11:RREB1:ATXN7L1* |
| YTAATTAA LHX3 01 | 193 | 3 | 4.33E-04 | 2.57E-02 | *RREB1:ATXN7L1:DMRTA1* |
| OCT1 03 | 233 | 3 | 7.48E-04 | 2.57E-02 | *SALL1:FBXO11:HDAC9* |
| CDPCR3HD 01 | 237 | 3 | 7.86E-04 | 2.57E-02 | *NEGR1:CLSTN2:ATXN7L1* |
| MEF2 03 | 237 | 3 | 7.86E-04 | 2.57E-02 | *FBXO11:PPP2R3A:HDAC9* |
| HNF6 Q6 | 238 | 3 | 7.96E-04 | 2.57E-02 | *SALL1:FBXO11:CLSTN2* |
| BRN2 01 | 241 | 3 | 8.25E-04 | 2.57E-02 | *SALL1:NPAS2:KCNMB2* |
| GATA3 01 | 245 | 3 | 8.65E-04 | 2.57E-02 | *SALL1:FBXO11:HDAC9* |
| S8 01 | 248 | 3 | 8.96E-04 | 2.57E-02 | *CLSTN2:RREB1:ATXN7L1* |
| HFH1 01 | 250 | 3 | 9.17E-04 | 2.57E-02 | *HMCN1:PPP2R3A:RREB1* |
| ISRE 01 | 252 | 3 | 9.38E-04 | 2.57E-02 | *HMCN1:FBXO11:ATXN7L1* |
| PAX2 01 | 59 | 2 | 9.47E-04 | 2.57E-02 | *NPAS2:HDAC9* |
| COUP DR1 Q6 | 253 | 3 | 9.49E-04 | 2.57E-02 | *MICAL2:SALL1:RREB1* |
| STAT1 03 | 253 | 3 | 9.49E-04 | 2.57E-02 | *SALL1:FBXO11:HDAC9* |
| E47 02 | 254 | 3 | 9.60E-04 | 2.57E-02 | *HMCN1:NPAS2:HDAC9* |
| PBX1 01 | 259 | 3 | 1.02E-03 | 2.57E-02 | *SALL1:FBXO11:HDAC9* |
| CP2 01 | 261 | 3 | 1.04E-03 | 2.57E-02 | *SALL1:CLSTN2:HDAC9* |
| OCT C | 266 | 3 | 1.10E-03 | 2.57E-02 | *SALL1:PPP2R3A:HDAC9* |
| ZIC1 01 | 266 | 3 | 1.10E-03 | 2.57E-02 | *NEGR1:SALL1:ATXN7L1* |
| MYCMAX B | 272 | 3 | 1.17E-03 | 2.64E-02 | *MICAL2:SALL1:SERPINF1* |
| **GWAS catalog (5)** | | | | | |
| Iris color (b* coordinate) | 28 | 3 | 1.31E-06 | 2.37E-03 | *OCA2:CLSTN2:HDAC9* |
| Asthma | 337 | 5 | 5.34E-06 | 4.36E-03 | *NEGR1:FBXO11:CLSTN2:HDAC9:DMRTA1* |
| Lymphocyte counts | 163 | 4 | 7.20E-06 | 4.36E-03 | *RREB1:HDAC9:ANK1:DMRTA1* |
| Post bronchodilator FEV1/FVC ratio | 199 | 4 | 1.58E-05 | 7.17E-03 | *GPR123:LRRC4C:OCA2:CLSTN2* |
| Moyamoya disease | 16 | 2 | 6.78E-05 | 2.46E-02 | *SPTLC3:HDAC9* |

## Table S7. Top genes with rare and low-frequency variants associated with SB in AI using extended model.

| **Chr** | **Positions** | **Gene** | **Variants** | **SNPs** | **%Rare** | ***p*-value** | **FDR** |
| --- | --- | --- | --- | --- | --- | --- | --- |
| 1 | 208062056-208073330 | *CD34* | Nonsyn | 9(12) | 2.70 | 1.23E-06 | 0.032 |
| 1 | 48694570-48713100 | *SLC5A9* | Nonsyn | 12(16) | 8.78 | 1.95E-06 | 0.032 |
| 1 | 48688376-48714188 | *SLC5A9* | Exonreg | 27(35) | 20.27 | 3.34E-06 | 0.032 |
| 19 | 35923858-35924757 | *AC002511.3* | Exonreg | 3(5) | 2.43 | 4.41E-06 | 0.032 |
| 1 | 29138936-29190138 | *OPRD1* | Exonreg | 3(7) | 3.11 | 4.53E-06 | 0.032 |
| 19 | 35418193-35435632 | *ZNF30* | Nonsyn | 15(21) | 8.51 | 4.58E-06 | 0.032 |
| 17 | 1673263-1680721 | *SERPINF1* | Nonsyn | 9(10) | 5.41 | 1.19E-05 | 0.069 |
| 17 | 1665330-1680847 | *SERPINF1* | Exonreg | 12(17) | 5.81 | 1.47E-05 | 0.069 |
| 2 | 74588717-74598791 | *DCTN1* | Nonsyn | 11(11) | 9.59 | 1.51E-05 | 0.069 |
| 21 | 45750089-45751822 | *C21orf2* | Nonsyn | 4(5) | 2.84 | 2.02E-05 | 0.083 |
| 9 | 98997810-99064425 | *HSD17B3* | Exonreg | 10(12) | 10.41 | 2.52E-05 | 0.095 |

## Table S8. Top pathways and gene sets with rare and low-frequency nonsynonymous variants associated with SB in AI using extended model.

| **Database** | **Pathway** | **SNPs** | **%Rare** | ***p*-value** | **FDR** |
| --- | --- | --- | --- | --- | --- |
| REACTOME | REGULATION OF GENE EXPRESSION BY HYPOXIA INDUCIBLE FACTOR | 82(91) | 49.5 | 6.36E-06 | 0.019 |
| KEGG | VASOPRESSIN REGULATED WATER REABSORPTION | 163(189) | 78.2 | 2.30E-05 | 0.024 |
| WP | VASOPRESSIN REGULATED WATER REABSORPTION | 164(190) | 78.8 | 2.65E-05 | 0.024 |
| REACTOME | CELLULAR HEXOSE TRANSPORT | 127(151) | 62.3 | 3.21E-05 | 0.024 |

## Table S9. Functional networks associated with genes whose rare variants were significantly associated with suicidal behaviors (FDR<0.1).

See Table 2. Three top distinct functional networks (bold font) are highlighted in Figure 3.

| Functional networks | FDR | Genes in network | Genes in genome |
| --- | --- | --- | --- |
| **vascular endothelial growth factor receptor signaling pathway** | 2.05E-04 | 4 | 30 |
| **angiogenesis** | 2.05E-04 | 7 | 298 |
| regulation of angiogenesis | 2.51E-04 | 6 | 203 |
| regulation of endothelial cell proliferation | 3.10E-04 | 5 | 112 |
| regulation of vasculature development | 3.10E-04 | 6 | 225 |
| endothelial cell proliferation | 3.80E-04 | 5 | 121 |
| epithelial cell proliferation | 5.33E-04 | 6 | 266 |
| **response to decreased oxygen levels** | 4.19E-03 | 5 | 208 |
| response to oxygen levels | 4.91E-03 | 5 | 220 |
| regulation of epithelial cell proliferation | 5.73E-03 | 5 | 232 |
| positive regulation of epithelial cell proliferation | 6.67E-03 | 4 | 107 |
| positive regulation of vasculature development | 8.15E-03 | 4 | 115 |
| cellular response to vascular endothelial growth factor stimulus | 1.21E-02 | 3 | 39 |
| response to hypoxia | 1.62E-02 | 4 | 147 |
| cellular response to decreased oxygen levels | 1.62E-02 | 4 | 147 |
| transmembrane receptor protein tyrosine kinase activity | 1.62E-02 | 3 | 45 |
| cellular response to oxygen levels | 2.13E-02 | 4 | 160 |
| hormone biosynthetic process | 2.35E-02 | 3 | 54 |
| regulation of chemotaxis | 2.83E-02 | 4 | 177 |
| regulation of vasculogenesis | 5.28E-02 | 2 | 11 |
| cell chemotaxis | 5.97E-02 | 4 | 220 |
| ganglion development | 6.68E-02 | 2 | 13 |
| transmembrane receptor protein kinase activity | 6.68E-02 | 3 | 83 |
| angiogenesis involved in wound healing | 8.04E-02 | 2 | 15 |
| cellular response to hypoxia | 8.04E-02 | 3 | 90 |
| endothelium development | 9.45E-02 | 3 | 103 |
| protein tyrosine kinase activity | 9.45E-02 | 3 | 102 |
| semaphorin-plexin signaling pathway | 9.45E-02 | 2 | 17 |
| regulation of positive chemotaxis | 9.45E-02 | 2 | 18 |
| positive chemotaxis | 9.45E-02 | 2 | 18 |
| positive regulation of cell migration | 9.45E-02 | 4 | 274 |
| positive regulation of chemotaxis | 9.94E-02 | 3 | 106 |

## Table S10a. Top genes with rare and low-frequency variants associated with SB-AUD in AI using the main model.

No gene has passed the genome-wide significant threshold after multiple testing correction.

| **Chr** | **Positions** | **Gene** | **Variants** | **SNPs**^1^ | **%Rare**^2^ | ***P*_AUD_**^3^ | ***P*_SB_**^3^ | ***P*_SB-AUD_**^4^ |
| --- | --- | --- | --- | --- | --- | --- | --- | --- |
| 1 | 208062056-208073330 | *CD34* | Nonsyn | 9(12) | 2.69 | 2.28E-02 | 3.55E-06 | 2.84E-05 |
| 2 | 74588717-74598791 | *DCTN1* | Nonsyn | 11(11) | 9.56 | 1.49E-02 | 1.73E-05 | 4.49E-05 |
| 2 | 30748587-30863108 | *LCLAT1* | Nonsyn | 3(5) | 0.81 | 4.10E-05 | 1.49E-02 | 7.54E-05 |
| 17 | 1481590-1518318 | *SLC43A2* | Nonsyn | 7(7) | 2.42 | 1.30E-02 | 6.19E-05 | 8.42E-05 |
| 15 | 81605656-81614811 | *STARD5* | Nonsyn | 3(3) | 5.65 | 2.43E-02 | 2.39E-05 | 9.01E-05 |
| 21 | 48018982-48025113 | *S100B* | Exonreg | 10(14) | 12.7 | 2.58E-03 | 1.01E-04 | 2.67E-05 |
| 5 | 31744189-31744551 | *RP11-5N11.5* | Exonreg | 5(5) | 4.2 | 2.37E-03 | 5.38E-04 | 7.85E-05 |
| 22 | 41631149-41636918 | *CHADL* | Exonreg | 5(9) | 1.5 | 1.38E-02 | 8.84E-05 | 1.11E-04 |
| 9 | 98177796-98189122 | *RP11-435O5.2* | Exonreg | 3(7) | 12.0 | 1.02E-02 | 2.64E-04 | 1.66E-04 |
| 11 | 125365285-125369367 | *AP000708.1* | Exonreg | 21(24) | 29.5 | 5.14E-04 | 6.26E-03 | 1.69E-04 |

^1^ Number of rare/low-frequency markers included in the test for each gene. The number in the parenthesis is the total number of SNPs of the same category on the gene.

^2^ The fraction of individuals that have at least one of the rare/low-frequency markers on the gene.

^3^ *P*_AUD_: *p-*value for AUD severity gene-based test; *P*_SB_: *p*-value for SB gene-based test.

^4^ *P*_SB-AUD_: *p*-value for meta-analysis of gene-based tests between SB and AUD severity.

## Table S10b. Top pathways with rare and low-frequency nonsynonymous variants associated with SB-AUD in AI using the main model.

No pathway has passed the genome-wide significant threshold after multiple testing correction.

| **Database** | **Pathway** | **SNPs** | **%Rare** | ***P*_AUD_** | ***P*_SB_** | ***P*_SB-AUD_** |
| --- | --- | --- | --- | --- | --- | --- |
| REACTOME | REGULATION OF GENE EXPRESSION BY HYPOXIA INDUCIBLE FACTOR | 83(91) | 49.5 | 3.13E-01 | 1.35E-05 | 1.83E-03 |
| KEGG | TAURINE AND HYPOTAURINE METABOLISM | 43(56) | 40.3 | 7.06E-04 | 1.58E-01 | 4.65E-03 |
| REACTOME | LINOLEIC ACID LA METABOLISM | 16(17) | 16.3 | 9.97E-02 | 1.89E-03 | 4.76E-03 |
| PID | HIF1A PATHWAY | 57(65) | 44.2 | 3.11E-02 | 1.04E-02 | 4.79E-03 |
| REACTOME | WAX AND PLASMALOGEN BIOSYNTHESIS | 17(18) | 18.2 | 8.59E-02 | 2.61E-03 | 4.94E-03 |

# **Supplementary Material and Method**

## S1. Participants

American Indian participants were recruited from eight geographically contiguous reservations with a total population of about 3,000 individuals. To be included in the study, participants had to be between the ages of 18 and 70 years, and mobile enough to be transported from their home to The Scripps Research Institute (TSRI). Participants were recruited using a combination of a venue-based method for sampling hard-to-reach populations [Kalton and Anderson, 1986; Muhib et al., 2001] and a respondent-driven procedure [Heckathorn, 2014] that has been described elsewhere [Gilder et al., 2004]. Approximately half of the participants were recruited using each method. A 10-25% rate of refusal using the venue method occurred depending on venue. Refusal rates were higher at tribal libraries and stores than health clinics and tribal halls or culture centers. The refusal rate in the respondent-driven procedure is not known. The protocol for the study was approved by the Institutional Review Board (IRB) of TSRI, and the board of the Indian Health Council, a tribal review group overseeing health issues for the reservations where the recruitment was undertaken. Written informed consent was obtained from each participant after the study was fully explained.

## S2. AUD severity phenotype

The SSAGA is a semi-structured, poly-diagnostic psychiatric interview that has undergone both reliability and validity testing based on DSM-IV [Bucholz et al., 1994; Hesselbrock et al., 1999]. It has also been used in another AI sample [Hesselbrock et al., 2000] [Hesselbrock et al., 2003], which collected information including demographics, psychiatric history, and symptoms of substance use disorders. Diagnoses of lifetime DSM-5 AUD (mild, moderate, or severe) were generated using the SSAGA. A research psychiatrist/addiction specialist made all best final diagnoses of AUD [Ehlers et al., 2004; Gilder et al., 2004]. In addition, the interview retrospectively asks about the occurrence of alcohol-related life events, and the age at which the problem first occurred, from which one of the main quantitative phenotypes for this study, the severity level of AUD, was derived.

Schuckit and colleagues [Schuckit et al., 1993] described measures of the clinical course of alcoholism based on the relative order of the appearance of major “alcohol-related life events”. The clinical course of AUD for the American Indian cohort was previously described [Ehlers et al., 2015; Ehlers et al., 2004]. The severity level of AUD was indexed by 36 alcohol-related life events (Table S1) in the clinical course of the disorder, with life events given a severity weight of 1 for events 1-12; 2 for 13-24; and 3 for 25-36. AUD severity was then calculated as the sum of the severity weights of the 36 life events [Peng et al., 2019].

## S3. Genotyping of American Indian cohort

The whole genome sequencing pipeline of the AI cohort was previously described [Bizon et al., 2014]. In brief: blood derived DNA was sequenced using Illumina low-coverage whole genome sequencing (LCWGS): as well as genotyped using an Affymetrix Exome1A chip. The pair-end sequencing was performed on HiSeq2000 sequencers (Illumina). About 80% of the samples had coverage between 3X and 12X: approximately evenly distributed. Reads from whole genome sequencing were aligned to the GRCh37/hg19 human reference genome using BMA: and realigned near indels with GATK [DePristo et al., 2011]. Variants were called using both GATK Unified Genotyper following the best practices for low-coverage samples [Van der Auwera et al., 2002] and the LD-aware variant caller Thunder [Li et al., 2011]. Imputation was carried out using the program Thunder. Qualities of variant calling were assessed through a comparison between the sequencing results to genotypes generated on the exome array for the same set of subjects. The assessment showed nearly all of common variants and a high percentage of rare variants in the samples were correctly called. The median concordance rate was 97.5%. The median false positive rate was 0.3% [Bizon et al., 2014]. The variant calling process resulted 23,550,342 genome-wide variants for 750 AI individuals.

Further quality control (QC) and filtering were performed as follows prior to genome-wide association analyses: removing variants with high missing rate (>5%), variants out of Hardy-Weinberg equilibrium (HWE) (*p*<1E-6), variants having high Mendel error rate (>5%), and removing individuals if missing >2% genotypes. For bivariate association analysis, variants having a MAF lower than 1% were excluded.

## S4. Rare and low frequency variant analysis

Rare variants are usually tested by aggregating them into groups. Two major classes of tests are typically used: the burden statistic and the sequence kernel association test (SKAT) statistic [Wu et al., 2011]. The burden test assumes that all rare variants being tested are causal and have the same direction of effects, while SKAT assumes that a small number of rare variants are causal and/or the effects may have mixed directions. Depending on the scenario of the individual group of variants tested, one statistic may be more powerful than the other. We thus analyzed the rare and low-frequency variants across the genome using a unified approach called SKAT-O that optimally combines a burden test and a non-burden SKAT [Lee et al., 2012] within a linear mixed model as implemented in EPACTS [Kang et al., 2010].

## S5. Pathway database used in rare-and-low-frequency variant test

We downloaded a collection of canonical pathways from the Molecular Signatures Database (MSigDB) version 7.5.1 [Liberzon et al., 2015; Subramanian et al., 2005]. The collection is comprised of pathways from multiple curated databases including BioCarta [Nishimura, 2001], KEGG [Kanehisa et al., 2016], the pathway interaction database (PID) [Schaefer et al., 2009], Reactome [Gillespie et al., 2022], and WikiPathways [Kutmon et al., 2016]. Each pathway is made of a gene set. The gene sets are canonical representations of a biological process compiled by domain experts. There are total of 2,981 pathway gene sets in this release. The file was downloaded from [GSEA](http://www.gsea-msigdb.org/gsea/msigdb/download_file.jsp?filePath=/msigdb/release/7.5.1/c2.cp.v7.5.1.symbols.gmt).

## S6. Meta-analysis of SB and AUD rare-and-low-frequency variant tests

To investigate the effects of rare and low-frequency variants on SB-AUD, we used a meta-analysis. Some of the rare variant analysis results on AUD severity were previously reported [Peng et al., 2019]. Since rare variant association tests on two traits were performed on the same cohort, the test statistics were correlated. We thus corrected this correlation prior to meta-analysis. For each set of gene- and pathway-based tests, summary statistics for SB and AUD severity were combined using the following procedure: 1) *p*-values were converted to Z-scores; 2) Z-scores from SB and AUD severity were de-correlated using a linear transformation that adjusts the joint distribution of the test statistics from the two tests [LeBlanc et al., 2018]; 3) de-correlated Z-scores were combined using Stouffer’s weighted Z-score method [Stouffer et al., 1949]; 4) the final Z-scores for SB-AUD were converted to *p*-values.

The Z-score decorrelation was carried out as the following [LeBlanc et al., 2018]. Let $\boldsymbol{Z}$ be a matrix with 2 rows and *m* columns where *m* is the number of genes (or pathways) tested. Each row in $\boldsymbol{Z}$ was the Z-scores from one of the two phenotypes. Let *r* be the correlation between the two phenotypes, *n*_1_ and *n*_2_ be the sample sizes for each phenotype, and *n*_c_ be the number of overlapping samples. The decorrelation transformation was defined as $\boldsymbol{Z}_{dc}=\boldsymbol{C}^{{-1}/2} \boldsymbol{Z}$, where $\boldsymbol{C}=\left[ \begin{matrix} 1 & \frac{n_{c} r}{\sqrt{n_{1}n_{2}}} \\ \frac{n_{c} r}{\sqrt{n_{1}n_{2}}} & 1 \end{matrix} \right]$.

## S7. Functional analysis with FUMA

The variants with *p*-value < 10^-6^ from the bivariate analysis were annotated with genes using SGAdviser [Pham et al., 2015]. The associated set of genes was then subjected to functional analysis. The same set of functional analysis was applied to the top genes (Table 2) from the rare variants analysis as well.

Functional enrichment analyses were performed using GENE2FUNC in FUMA version 1.5.1, where enriched biological functions or pathways were extracted by testing against gene sets from the molecular signatures database (MsigDB) [Liberzon et al., 2011] and WikiPathways [Kutmon et al., 2016] using hypergeometric tests [Watanabe et al., 2017]. The tissue-specific differential gene expression test was conducted against all genes across genomes that exhibited significantly increased or decreased expression levels in a certain tissue sample compared to all other samples. The analysis utilized tissue-specific transcriptome data across 54 tissue types from GTEx v8 [GTEx Consortium, 2015].

# **Supplementary Reference**

Bizon C, Spiegel M, Chasse SA, Gizer IR, Li Y, Malc EP, Mieczkowski PA, Sailsbery JK, Wang X, Ehlers CL, Wilhelmsen KC. 2014. Variant calling in low-coverage whole genome sequencing of a Native American population sample. BMC Genomics **15**:85.

Bucholz KK, Cadoret R, Cloninger CR, Dinwiddie SH, Hesselbrock VM, Nurnberger JI, Jr., Reich T, Schmidt I, Schuckit MA. 1994. A new, semi-structured psychiatric interview for use in genetic linkage studies: a report on the reliability of the SSAGA. Journal of Studies on Alcohol **55**:149-158.

DePristo MA, Banks E, Poplin R, Garimella KV, Maguire JR, Hartl C, Philippakis AA, del Angel G, Rivas MA, Hanna M, McKenna A, Fennell TJ, Kernytsky AM, Sivachenko AY, Cibulskis K, Gabriel SB, Altshuler D, Daly MJ. 2011. A framework for variation discovery and genotyping using next-generation DNA sequencing data. Nature Genetics **43**:491-498.

Ehlers CL, Stouffer GM, Corey L, Gilder DA. 2015. The clinical course of DSM-5 alcohol use disorders in young adult native and Mexican Americans. The American Journal on Addictions **24**:713-721.

Ehlers CL, Wall TL, Betancourt M, Gilder DA. 2004. The clinical course of alcoholism in 243 Mission Indians. American Journal of Psychiatry **161**:1204-1210.

Gilder DA, Wall TL, Ehlers CL. 2004. Comorbidity of select anxiety and affective disorders with alcohol dependence in southwest California Indians. Alcohol Clin Exp Res **28**:1805-1813.

Gillespie M, Jassal B, Stephan R, Milacic M, Rothfels K, Senff-Ribeiro A, Griss J, Sevilla C, Matthews L, Gong C, Deng C, Varusai T, Ragueneau E, Haider Y, May B, Shamovsky V, Weiser J, Brunson T, Sanati N, Beckman L, Shao X, Fabregat A, Sidiropoulos K, Murillo J, Viteri G, Cook J, Shorser S, Bader G, Demir E, Sander C, Haw R, Wu G, Stein L, Hermjakob H, D’Eustachio P. 2022. The reactome pathway knowledgebase 2022. Nucleic Acids Research **50**:D687-D692.

GTEx Consortium T. 2015. The Genotype-Tissue Expression (GTEx) pilot analysis: Multitissue gene regulation in humans. Science **348**:648-660.

Heckathorn DD. 2014. Respondent-Driven Sampling: A New Approach to the Study of Hidden Populations*. Social Problems **44**:174-199.

Hesselbrock M, Easton C, Bucholz KK, Schuckit M, Hesselbrock V. 1999. A validity study of the SSAGA--a comparison with the SCAN. Addiction **94**:1361-1370.

Hesselbrock MN, Hesselbrock VM, Segal B, Schuckit MA, Bucholz K. 2003. Ethnicity and Psychiatric Comorbidity Among Alcohol-Dependent Persons Who Receive Inpatient Treatment: African Americans, Alaska Natives, Caucasians, and Hispanics. Alcohol: Clinical and Experimental Research **27**:1368-1373.

Hesselbrock VM, Segal B, Hesselbrock MN. 2000. Alcohol dependence among Alaska Natives entering alcoholism treatment: a gender comparison. Journal of Studies on Alcohol **61**:150-156.

Kalton G, Anderson DW. 1986. Sampling Rare Populations. Journal of the Royal Statistical Society: Series A (General) **149**:65-82.

Kanehisa M, Sato Y, Kawashima M, Furumichi M, Tanabe M. 2016. KEGG as a reference resource for gene and protein annotation. Nucleic Acids Research **44**:D457-D462.

Kang HM, Sul JH, Service SK, Zaitlen NA, Kong S-Y, Freimer NB, Sabatti C, Eskin E. 2010. Variance component model to account for sample structure in genome-wide association studies. Nature Genetics **42**:348-354.

Kutmon M, Riutta A, Nunes N, Hanspers K, Willighagen Egon L, Bohler A, Mélius J, Waagmeester A, Sinha Sravanthi R, Miller R, Coort SL, Cirillo E, Smeets B, Evelo Chris T, Pico AR. 2016. WikiPathways: capturing the full diversity of pathway knowledge. Nucleic Acids Research **44**:D488-D494.

LeBlanc M, Zuber V, Thompson WK, Andreassen OA, Frigessi A, Andreassen BK, Schizophrenia, Bipolar Disorder Working Groups of the Psychiatric Genomics C. 2018. A correction for sample overlap in genome-wide association studies in a polygenic pleiotropy-informed framework. BMC Genomics **19**:494.

Lee S, Emond MJ, Bamshad MJ, Barnes KC, Rieder MJ, Nickerson DA, Team NGESPELP, Christiani DC, Wurfel MM, Lin X. 2012. Optimal unified approach for rare-variant association testing with application to small-sample case-control whole-exome sequencing studies. American journal of human genetics **91**:224-237.

Li Y, Sidore C, Kang HM, Boehnke M, Abecasis GR. 2011. Low-coverage sequencing: implications for design of complex trait association studies. Genome Research **21**:940-951.

Liberzon A, Birger C, Thorvaldsdóttir H, Ghandi M, Mesirov Jill P, Tamayo P. 2015. The Molecular Signatures Database Hallmark Gene Set Collection. Cell Systems **1**:417-425.

Liberzon A, Subramanian A, Pinchback R, Thorvaldsdóttir H, Tamayo P, Mesirov JP. 2011. Molecular signatures database (MSigDB) 3.0. Bioinformatics **27**:1739-1740.

Muhib FB, Lin LS, Stueve A, Miller RL, Ford WL, Johnson WD, Smith PJ. 2001. A Venue-Based Method for Sampling Hard-to-Reach Populations. Public Health Reports **116**:216-222.

Nishimura D. 2001. BioCarta. Biotech Software & Internet Report **2**:117-120.

Peng Q, Bizon C, Gizer IR, Wilhelmsen KC, Ehlers CL. 2019. Genetic loci for alcohol-related life events and substance-induced affective symptoms: indexing the “dark side” of addiction. Translational Psychiatry **9**:71.

Pham PH, Shipman WJ, Erikson GA, Schork NJ, Torkamani A. 2015. Scripps Genome ADVISER: Annotation and Distributed Variant Interpretation SERver. PLoS One **10**:e0116815.

Purcell S, Cherny SS, Sham PC. 2003. Genetic Power Calculator: design of linkage and association genetic mapping studies of complex traits. Bioinformatics **19**:149-150.

Schaefer CF, Anthony K, Krupa S, Buchoff J, Day M, Hannay T, Buetow KH. 2009. PID: the Pathway Interaction Database. Nucleic Acids Research **37**:D674-D679.

Schuckit MA, Smith TL, Anthenelli R, Irwin M. 1993. Clinical course of alcoholism in 636 male inpatients. American Journal of Psychiatry **150**:786-792.

Sham PC, Purcell SM. 2014. Statistical power and significance testing in large-scale genetic studies. Nat Rev Genet **15**:335-346.

Stouffer S, DeVinney L, Suchmen E. 1949. The American soldier: Adjustment during army life. Princeton, US: Princeton University Press.

Subramanian A, Tamayo P, Mootha VK, Mukherjee S, Ebert BL, Gillette MA, Paulovich A, Pomeroy SL, Golub TR, Lander ES, Mesirov JP. 2005. Gene set enrichment analysis: A knowledge-based approach for interpreting genome-wide expression profiles. Proceedings of the National Academy of Sciences **102**:15545.

Van der Auwera GA, Carneiro MO, Hartl C, Poplin R, del Angel G, Levy-Moonshine A, Jordan T, Shakir K, Roazen D, Thibault J, Banks E, Garimella KV, Altshuler D, Gabriel S, DePristo MA. 2002. From FastQ Data to High-Confidence Variant Calls: The Genome Analysis Toolkit Best Practices Pipeline. Current Protocols in Bioinformatics: John Wiley & Sons, Inc.

Watanabe K, Taskesen E, van Bochoven A, Posthuma D. 2017. Functional mapping and annotation of genetic associations with FUMA. Nature Communications **8**:1826.

Wu MC, Lee S, Cai T, Li Y, Boehnke M, Lin X. 2011. Rare-variant association testing for sequencing data with the sequence kernel association test. The American Journal of Human Genetics **89**:82-93.
